# Supplementary material for: Caregivers’ perception of risk for malaria, helminth infection and malaria-helminth co-infection among children living in urban and rural settings of Senegal: A qualitative study
Source: PLOS Glob Public Health. 2022 Aug 12;2(8):e0000525. doi: 10.1371/journal.pgph.0000525 (PMC10021862; doi:10.1371/journal.pgph.0000525)
Supplement: S1 Table — (DOCX) [file pgph.0000525.s001.docx]

S1 Table: Interview guide used to collect qualitative data from the caregivers

**Caregivers’ perception of risk factors for malaria-helminthic co-infections among Senegelese children**

Greet the parents and introduce the study that it is aimed to understand the perceptions of mothers/care-givers about what may make their children have malaria with worms at the same time. Please ensure you obtain consent from the mother/care-giver to interview her and to record the interview before starting the interview.

| **Component** | **Interview guide** |  |
| --- | --- | --- |
| **Risk perception for malaria** | **Now I’m going to ask you some questions about malaria:**   - “Tell me about your sleeping habits as they relate to bednets.” **Then let them tell you about it first. If they don’t tell you how often, etc., then probe** “How often do you sleep under a bednet? How often do your children sleep under a bednet? Does anyone in your family not sleep under a bednet? Why not?” - **For the second question, first ask**: “Tell me about your bednet.” **Let them speak. Then probe**: “Is it in good order?” **Let them speak.** “Are there any holes or is it torn anywhere?” - “Tell me how you care for your bednet.” **Let them speak. Probe**: “Do you wash it? How and how often? Do you treat it? How and how often?” | - Explore further to know the practice of sleeping under the bednet. e.g. whether the child or the caregivers slept under a bednet the night before interview, almost every night, 3-4 nights per week, 1-2 nights per week, 1-3 nights per month, less than once a month, never. - Find out the reason for either the regular or non-practice of sleeping under bednet. - Apart from bednet, are there other methods used by the caregivers to protect themselves and the child (ren) from mosquito bites e.g. indoor spraying with insecticides either by government officers or personally |
| **Household structure** | **Now, I am going to ask you a couple of questions about the structure of your home:**  Tell me about your house – how was it constructed ?  **Then from there, you can probe with the following specific questions**   - What are the walls of your house made of ? - What is the roof of your house made of? - Is there a gap between the walls of your house and the roof - Does your house have nets on the windows and doors ? | - Explore further to know if the walls were made of mud and poles, cement blocks, corrugated iron, concrete , mud bricks, burnt bricks, wood, etc ? - Explore further to know if the roof of the house is made of corrugated iron, grass, etc ? |
| **Health-seeking behaviour for malaria** | **Tell me about how malaria has affected your child(ren).**   - Has your child(ren) got malaria before ? Tell me about that experience. - How often do they get malaria ? When was the last time ? - Tell me about how you treated your child for malaria. What did you do ? **Then you can probe** : Did you go the health centre ? Did you go to a traditional healer ? | - Explore how malaria was treated in the child:   self-treatment, neighbors/friends, medicine vendors,  traditional healers, primary health unit, private clinic , etc   - Explore the types of care given: drugs only, cold bath, herbal concoction, hot fomentation, massage, scarifications, spiritual oils or incantations, combination of two or more listed here, etc |
| **Perception and opinions regarding water-related services** | |  |
| Availability and accessibility of water sources | **Tell me about water in your community. Then probe further with the questions below :**   - How would you describe the availability of water in your community? Probe : Do you ever face the problems of not having enough water to drink/cook/clean/bathe with ? How often ? Are there certain times when water is easier or harder to have ? - Where does water in your community come from? Can you tell me all the sources of water in your community ? Probe : is there running water ? wells ? common taps, borehole etc.) - Where are these water sources located ? How long does it take you to get water when you need it ? Does this affect your daily activities at all ? - Tell me about the quality of water in your community. Can you describe the taste and cleanliness of the water to me?   **You just told me about water in your community, but now I want to know about the water in your household specifically and how you and your family use water**:   - What are some of the ways you use this water? Are there certain things you don’t do with the water from your community? - **Then probe on specific use**s: Is there anything you do to the water before using it ? (Probe : boil water boost, use of bleach, decantation technique or other methods (including traditional methods etc.) | - The interviewers will probe further based on the answers provided by the mothers/care-givers |
| **Personal experience** | |  |
|  | **Earlier you mentioned all sources of water in your community. Which source of water do you use for your household? (Remind the participant on the sources of water, the location of the source, collection time)**   - Do you use any other sources ? - What are the reasons for this choice?/Why this source ? - Is there anything that makes it difficult to access this water source ? What is that ? What are the things that make it difficult to access this water? - How do you store water in your house ? |  |
| **Hygiene and sanitation practices** | |  |
|  | **Now, I am going to ask you a couple of questions about the toilets in your house:**  Can you tell me about the toilets in your household? And then probe: what is the structure of your toilets like? How do you take care of the toilets?   - Are these toilets shared with other households? Probe: how many households use these toilets? - How is the stool of your children managed? Probe: Who is responsible for managing the stool of your children? Where is the stool disposed? - What kind of toilet pot is used for defecation of your children? (reminder on the use of toilet pots, latrines etc.) Probe: How do you take care of toilet pots of your children (**reminder on where the faeces are thrown, how the toilet pots are cleaned etc**.) |  |
| **Practices related to hand washing** | |  |
| Personal experience | **Now, I want to ask you a few questions about handwashing**  How would you describe the handwashing practices in your community?   - Have you had any health education on the importance of hand washing? - What is your opinion about handwashing practices in your community? - How do you wash your hands? (**Reminder on the products used – soap, detergent products, water etc.?)**   - Can you indicate when to wash hands when cooking?   - Can you indicate when to wash your hands when using the toilet?   - Can you indicate when to wash your hands when feeding your children? | • The interviewer will verify the hand washing facilities at the location indicated by the respondent regarding the use of the toilet  • The interviewer will verify the hand washing facilities at the location indicated by the respondent regarding the use of the toilet   - The interviewer will verify the hand washing facilities at the location indicated by the respondent regarding the use of the toilet |
| Practices related to swimming and walking barefooted | - Is there a river, stream or pond near your house ? Tell me about it. Probe further - How far or close is the river, stream or pond to your house ? How long does it take to walk to the river/stream/pond ? - What activities take place at this river/stream/pond ? - Probe : Do you go there to wash clothes or items ? Do you go there to swim? - How often do you take children to wash or swim in the river, stream ? - Have you ever swum in the river or stream with your child(ren) ? - Do you know if your child(ren) swim in the river or stream ? If so, how often ? - Have you heard of anyone who has drowned in the river whilst swimming before ? How frequent is drowning in the river in your community ? Probe further. - Tell me about the shoe-wearing practices of your children. Probe further on the type of shoes and whether the children wear shoes frequently and why ? |  |
| Knowledge and perception about malaria-helminth co-infection | What are some common illnesses children may have? Are there any illnesses that can happen to a child at the same time? What are these illnesses?  Do you know your children can have malaria and worms together at the same time?  What are your thoughts about a child having malaria and worms at the same time? Does this happen to your children? Is it common?” And then ask specifically if it has happened to their child or if they thought it has happened.  What do you think could make a child get both malaria and worms together at the same time?  Are there any special signs or symptoms that a child who has both malaria and worms show? Are these symptoms or signs different if they only have malaria or worms separately? How could you tell a child has both malaria and worms at the same time?”   - What are some of the ways you think the problems of having both malaria and worms together can be prevented in children ? - What are your thoughts on medicines for worms or malaria prevention ? Probe - Have you allowed your children to be dewormed or given malaria prevention drugs before ? If yes, when was this ? Where did the medicines come from (government, health centres)   Do you think these drugs are useful to prevent your children from becoming sick with both malaria and worms together ? | Probe further the mother/care-giver’s perception about the need for regular deworming of the children |
| **Recommendations** | |  |
|  | What would you tell other mothers to help them prevent malaria and worms in their own children? What would you advise government to do to help prevent malaria and worms in children in your community?” |  |

Thank you very much for speaking with us.
