# Supplementary material for: Caregivers’ perception of risk for malaria, helminth infection and malaria-helminth co-infection among children living in urban and rural settings of Senegal: A qualitative study
Source: PLOS Glob Public Health. 2022 Aug 12;2(8):e0000525. doi: 10.1371/journal.pgph.0000525 (PMC10021862; doi:10.1371/journal.pgph.0000525)
Supplement: Attachment — Submitted filename: RESPONSE SHEET_PGPH-D-22-00478.doc [file pgph.0000525.s002.doc]

13 May 2022

Dear Editor,

Revision of manuscript PGPH-D-22-00478

Caregivers' perception of risk for malaria, helminth infection and malaria-helminth co-infection among children living in urban and rural settings of Senegal: a qualitative study.

Thank you for sharing with us the reviewers’ comments on the above named manuscript. We are pleased that the Editors feel the manuscript has merit; and we have carefully taken the editorial and reviewers’ comments into account in the revision of the manuscript. We describe below how we have responded to specific comments.

We have addressed the concerns from the reviewers and editors, and hope this paper is now acceptable for publication in your journal.

Yours sincerely,

Muhammed Afolabi

On behalf of all co-authors

**Reviewers' comments:**

Journal Requirements:

1. Please provide additional details regarding participant consent. In the ethics statement in the Methods and online submission information, please ensure that you have specified whether: 1) whether the ethics committee approved the verbal/oral consent procedure, 2) why written consent could not be obtained, and 3) how verbal/oral consent was recorded. If your study included minors, please state whether you obtained consent from parents or guardians in these cases. If the need for consent was waived by the ethics committee, please include this information.

*Response: As requested we have included additional details regarding the participant consent in the ethics statement in the Methods section of the manuscript and online submission entry. On page 11, lines 250-260, we added ‘Given that the study was non-interventional and its implementation posed little or risk to the study participants, verbal consent was considered sufficient for this study. The Ethics Committees approved the verbal consent procedure for this study. The detailed information about the study was provided to a potential study participant by a trained research assistant, after the participant’s concerns and questions were satisfactorily addressed, they signified consent to participate in the study orally. This process of verbal consenting was documented by the research assistant in a dedicated consent register.’*

*The study interviewed caregivers who had attained legal age of consent, we did not enrol any minors into our study*

2. Please provide an Author Summary. This should appear in your manuscript between the Abstract (if applicable) and the Introduction, and should be 150–200 words long. The aim should be to make your findings accessible to a wide audience that includes both scientists and non-scientists. Sample summaries can be found on our website under Submission Guidelines: [https://journals.plos.org/globalpublichealth/s/submission-guidelines#loc-parts-of-a-submission](https://journals.plos.org/globalpublichealth/s/submission-guidelines#loc-parts-of-a-submission)

*Response: We have provided an author summary, as requested*

3. Please amend your Data Availability Statement and indicate where the data may be found

*Response: We have amended the data availability statement accordingly and indicated that the data would be provided by the first author following written request.*

**Editorial comments**

4. The authors need to clearly articulate the justification for conducting the study and how their findings contribute to the current scholarship

*Response: On page 5, lines 105-109 of the annotated manuscript, we clearly articulated the justification for conducting this study. We stated that…. ‘However, very few studies ^15,17^ have investigated the caregivers’ perception to the risk of dual or multiple infections such as malaria and worms. Understanding the importance of malaria-helminth coinfection and its influence on health-seeking behaviour from the caregivers’ perspectives is critical to the development and uptake of integrated approaches to prevention and treatment to the co-infection.*

*On page 22, lines 580-584, we discussed how the findings contribute to the current scholarship. We stated…. ‘The findings of this study underscore the need to promote awareness about the risk and complications of malaria-helminth co-infections in children. This step would assist in addressing the caregivers’ misconceptions about the co-infection and may improve their uptake of the strategic interventions developed to achieve control and elimination of malaria and helminth co-infection.’*

5. The authors need to discuss the theoretical framework underpinning their study. Was it an Ethnographic study or was it based on grounded theory or was it phenomenological? If no theoretical framework was used, it would be good for the authors to explain why. Also, it would be good attached a copy of the interview guide.

*Response: The theoretical framework underpinning this study was extensively discussed on page 5, lines 110-121. We stated…. ‘Given that the Health Belief Model (HBM) is one of the most important health behavioural theories^18^, we postulated a framework for this study on a central concept in the HBM as the ‘perceived susceptibility’, which refers to the perceived chance of acquiring a condition. The ‘perceived susceptibility’ and the ‘perceived severity’ leads to the formation of ‘perceived barrier’ of a certain condition. The likelihood of performing a certain health behaviour is directly linked to the ‘perceived threat’, the ‘perceived benefits’ and ‘barriers’ of the suggested behaviour change, the self-efficacy and the cues to action.^18,19^*

*Guided by the principles of HBM, we conducted this study to understand from the caregivers’ point of views as well as the behavioural and social risk factors (e.g. household sanitation and hand-washing practices) promoting malaria-helminth co-infection among pre-school and school-aged children living in the endemic rural and urban communities in Senegal. Although, our primary objective was to ascertain what the caregivers knew about malaria-helminth co-infection, we needed first to consider what they perceived to be the risks for malaria and helminths before exploring their perceptions on the co-infection involving malaria and helminths.*

*This study is an ethnographic study given that it employed multiple qualitative tools such as structured interviews and participant observations to understand the perception and behaviours of the caregivers about the risk of their children for malaria-helminth co-infections.*

*As requested, a copy of the interview guide used in this study has been attached to this submission as a supplementary material*

6. To ensure the validity of their findings, the authors need to discuss the trustworthiness of their study based on the following themes credibility, dependability, confirmability, transferability, and reflexivity.

*Response: We have discussed the trustworthiness of the study based on credibility, dependability, confirmability, transferability and reflexivity themes. On pages 9-10, lines 217-245, we added ‘*

*Study Trustworthiness: In line with trustworthiness criteria created by Lincoln and Guba^24^, we ensured credibility of the study by prolonged engagements, persistent observations, data collection triangulations, and researcher triangulations. Through an iterative process of listening, discussing, and re-listening, the team identified and consensually validated emerging themes and appended segments of dialogue supporting the proposed themes. We stopped interviews when saturation was reached (no new themes were identified). The team systematically reviewed the themes and sorted them into content domains. We used an analytic matrix to identify patterns and connections amongst the domains. Two of us not involved in the qualitative coding process (MA and IAM) audited the analytic matrix, choice of quotes, and thematic analysis.*

*The team members checked the descriptions of key phenomena and themes which emerged from the data analysis with the study participants and requested them to verify the accuracy or consistency with their perspectives. These activities supported the validity and transferability of our study findings.*

*We kept robust records of the raw data, field notes, transcripts, and a reflexive journal from this study and this helped us to seamlessly systemize, relate, and cross reference data. We maintained an audit trail that documented evidence of the decisions and choices that we made regarding the theoretical and methodological issues throughout the study period. These steps supported the dependability of our study.^25^*

*To achieve confirmability^25^, we highlighted the rationales for theoretical, methodological, and analytical choices throughout the entire study and this supported that the interpretations and findings were derived from the data collected from the study participants.*

*Reflexivity^25^ was maintained by the research team through the analysis and writing by recording, discussing and challenging established assumptions. In addition, MOA, NMS and AD kept reflexive diaries. The first author observed the interviews and discussion groups. He was not known to the participants of this research prior to undertaking the study as a medical doctor who had first-hand personal experience in the subject of discussion. Whilst it was useful to know (from his own background) what the participants were talking about medically (and in terms of detecting items of significance), as a researcher he made conscious efforts not to accept potentially common assumptions at face value.*

7. Please address reviewer 2 comments in the attached PDF document.

*Response: We have addressed all the comments raised by Reviewers 2 and updated the relevant sections of the manuscript accordingly.*

*It is instructive to state that we employed purposive sampling technique which is widely used in qualitative research for the identification and selection of information-rich cases for the most effective use of limited resources. Purposive sampling involves identifying and selecting individuals or groups of individuals that are especially knowledgeable about or experienced with a phenomenon of interest* (*Cresswell JW, Plano Clark VL. Designing and conducting mixed method research. 2nd Sage; Thousand Oaks, CA: 2011). In the context of our study, we purposively enrolled primary caregivers as our study participants; hence, there was no requirement to state whether the sample size was statistically valid because sample size is not calculated statistically a priori in qualitative research.*

*Similarly, given that quantitative data were not collected in this purely qualitative study, confirming that no formal quantitative analysis was done, it was not practicable to quantify the proportion of responses provided by the study participants. This is an acceptable standard approach in reporting qualitative research (O'Brien BC, Harris IB, Beckman TJ, Reed DA, Cook DA. Standards for reporting qualitative research: a synthesis of recommendations. Academic medicine: journal of the Association of American Medical Colleges. 2014;89(9):1245-51.)*

**Reviewers’ comments**

***Reviewer #1:***

8. The author need to be concise on what was the aim of the study, what were the study findings. Currently, the manuscript has ambiguity who was interviewed (caregivers versus the respondents) Observational data is more subjective and it is skewed to the interpretation of the observer.

*Response: We have clearly articulated the aim of the study as well as abundantly highlighted the study findings succinctly in the relevant section of this manuscript. On page 6, lines 117-120 of the annotated manuscript, we stated clearly that…….’we conducted this study to understand from the caregivers’ point of views as well as the behavioural and social risk factors (e.g. household sanitation and hand-washing practices) promoting malaria-helminth co-infection among pre-school and school-aged children living in the endemic rural and urban communities in Senegal’.*

*The study findings were also concisely summarised on page 22-23, lines 580-586 as follows: ……majority of the caregivers in our study demonstrated a high perception of risks as well as acceptable health-seeking behaviour for malaria, but low risk perception and misconceptions about the causative factors of worms and malaria-helminth co-infection. The findings of this study underscore the need to promote awareness about the risk and complications of malaria-helminth co-infections in children. This step would assist in addressing the caregivers’ misconceptions about the co-infection and may improve their uptake of the strategic interventions developed to achieve control and elimination of malaria and helminth co-infection’*

*Furthermore, it is an acceptable practice in qualitative research to use ‘respondents’ to describe ‘study participants’ and this does not in any way introduces any ambiguity to the fact that the caregivers were the study participants who responded to the questions posed to them during the qualitative interviews conducted in this study.*

9. Was the author determining knowledge of Malaria, helminths and co-infection or the perception of care givers to the severity of these infections? In the current format, the manuscript has no new information that can be published.

*Response: We respectfully disagree with the comment of the reviewer that this manuscript has no new information that can be published. As clearly stated above, our primary study objective was to determine the risk perception of the caregivers to malaria-helminth co-infections among their children. However, to ensure the caregivers understood the concept of the co-infection, we started by assessing their risk perception on malaria and helminths. These were abundantly clear in the last paragraph of the introduction of our manuscript.*

*As demonstrated in many parts of the document, our manuscript clearly contain new, useful information that contributes to expanding the limited literature about caregivers’ perception of risk of children to malaria-helminth co-infection.*

***Reviewer #2:***

10. The manuscript on “Caregivers’ perception of risk for malaria, helminth infection and malaria-helminth co-infection among children living in urban and rural settings of Senegal: a qualitative study” by Afolabi et al. attempts to qualitatively address an issue of public health significance. The purported findings attempt to underscore the need to promote caregivers’ awareness about the existence and risk of malaria-helminth co-infection in children. This is very relevant in shaping control policies on malaria and helminth infections in the said settings and other endemic areas especially where poverty and poor sanitary conditions are the norms. The authors in their paper have presented a narrative of their observations without any statistical analysis to back their findings. I acknowledge it is a qualitative study that notwithstanding the authors would have quantify the responses of the participants to carry out some statistical analysis to back their findings.

*Response: We thank and appreciate this reviewer for their kind and objective feedback on our manuscript. However, as stated in responses to Q7 above, this is a purely qualitative study with no quantitative data collected, hence no formal quantitative analysis was done, thereby making quantification of the proportion of responses provided by the study participants to be impractical. This is an acceptable standard approach in reporting qualitative research (O'Brien BC, Harris IB, Beckman TJ, Reed DA, Cook DA. Standards for reporting qualitative research: a synthesis of recommendations. Academic Medicine: Journal of the Association of American Medical Colleges. 2014;89(9):1245-51).*

11. Secondly, participants in the study have not been adequately described for the readers to relate to the respondents and how the authors arrived at the purposive sampling of a total of100 participants (50 from each study area).

*Response: On pages 6-7, lines 140-149, we added the description of the study population, as follows:*

*Study population: The study population consisted of primary caregivers of children aged 1-14 years who were vulnerable to malaria-helminth co-infection in Diourbel and Saraya districts of Senegal. In Saraya site, the primary caregivers were the parents and guardians of these children. In Diourbel site, pre-school and school aged children lived as full boarders in Koranic schools (also called ‘dahras’) where they received Arabic and Islamic teachings. The primary caregivers of the children learning in these schools were Koranic teachers, heads of Koranic schools and their wives. Older Koranic students (Supervising Talibé) were also appointed by the heads of the Koranic schools to supervise and serve as caregivers to the younger students in the schools.*

*On page 7, lines 156-171, we have updated the sample strategy, as follows:*

*Given that caregivers’ perceptions were previously shown to be related to gender, age, prior experience and place of domicile^15,17^, we purposely selected respondents with different backgrounds. This sampling strategy led to the diversity of gender, age, prior experience and place of domicile, whether in a rural or an urban setting. Purposive sampling was directed towards achieving maximum variation in age and gender, using a snowball approach: ‘a non-probabilistic form of sampling in which persons initially chosen for the sample are used as informants to locate other persons having necessary characteristics making them eligible for the sample.^23^ A purposive sampling method was used to recruit 50 participants across the three villages in Saraya district. These included 30 mothers, 10 fathers, 10 grandmothers who were primary caregivers of pre-school and school aged children. Similarly, we randomly selected 50 participants who were Koranic teachers and leaders of the Koranic schools (ouztaz) and were also primary caregivers for the Koranic students (talibés) in these schools (dahras). Sample size was estimated to be sufficient based on the principle of theoretical saturation^23^ and based on our previous experience with this methodology, we expected to reach saturation with approximately 50 interviews per group.*

*Furthermore, as discussed in the responses to Q7, we employed purposive sampling technique to recruit equal number of caregivers in each of the study sited who were the study participants in this research. Purposive sampling is widely used in qualitative research for the identification and selection of information-rich cases for the most effective use of limited resources. The sampling involves identifying and selecting individuals or groups of individuals that are especially knowledgeable about or experienced with a phenomenon of interest (Cresswell JW, Plano Clark VL. Designing and conducting mixed method research. 2nd Sage; Thousand Oaks, CA: 2011). In the context of our study, we purposively enrolled primary caregivers as our study participants, hence there was no requirement to state whether the sample size was statistically valid because a sample size calculation is not required in qualitative research.*

12. The intended audience would barely relate to the description of the housing structure and other unique set up described in the paper which is crucial to the understanding of the context if a graphical representation of these structures and map designating the various study sites are not included.

*Response: We have provided the maps of the study sites and the pictures of houses and other structures in the settings described in the paper in Figures 1, 2 and 3 on pages 26 and 27.*

13. There are several lapses in the paper that makes repetitiveness of the study and the assertion of the findings practically impossible. I have made several comments on the paper for suggested improvement if the authors deem in necessary to publish in a journal of such a repute.

*Response: We have addressed all comments raised by the reviewer accordingly and we believe the manuscript should be acceptable for publication in this journal. It is important to reiterate that a qualitative study does not statutorily require to have a formal quantitative analysis and quantification of responses by the study participants. This is in line with the acceptable reporting guidelines for qualitative research. (O'Brien BC, Harris IB, Beckman TJ, Reed DA, Cook DA. Standards for reporting qualitative research: a synthesis of recommendations. Academic Medicine: Journal of the Association of American Medical Colleges. 2014;89(9):1245-51)*
