# Supplementary material for: Caregivers’ perception of risk for malaria, helminth infection and malaria-helminth co-infection among children living in urban and rural settings of Senegal: A qualitative study
Source: PLOS Glob Public Health. 2022 Aug 12;2(8):e0000525. doi: 10.1371/journal.pgph.0000525 (PMC10021862; doi:10.1371/journal.pgph.0000525)
Supplement: Attachment — Submitted filename: RESPONSE SHEET_PGPH-D-22-00478R1.doc [file pgph.0000525.s003.doc]

18 July 2022

Dear Editor,

Revision of manuscript PGPH-D-22-00478R1: Caregivers' perception of risk for malaria, helminth infection and malaria-helminth co-infection among children living in urban and rural settings of Senegal: a qualitative study.

Thank you for sharing the second round of the reviewers’ comments on the above named manuscript. We do not agree with many of the reviewers’ submissions on this manuscript. This is because we have good reasons to believe that the reviewers’ comments and submissions reflect a phenomenon where a qualitative study is being reviewed from the prisms of research concepts underpinning quantitative studies and/or basic medical sciences, whereas qualitative research has its unique concepts of implementation and style of reporting. It would therefore be great if this manuscript could benefit from the review of an experienced social scientist or a medical anthropologist.

We highlight below our rebuttal and describe how we have responded to other specific comments from the reviewers.

Yours sincerely,

Muhammed Afolabi

On behalf of all co-authors

**Reviewers' comments:**

Reviewer #1: Title: It should be trimmed down to "Caregivers' perception of risk of malaria and Soil transmitted helminths. It should not be pegged on either mono-infection or co-infection.

*Response: Trimming down the title as suggested by the reviewer will distort the quintessence of the study and will not accurately represent the findings of the study reported in this manuscript. This study did not only focus on malaria and soil-transmitted helminth (STH) infections, but also on schistosomiasis. Given that helminths defined as parasitic worms are morphologically classified into trematodes (flukes – with schistosomes belonging to this class); cestodes (tapeworms) and nematodes (roundworms) - STH cuts across the two classes, we are of the opinion that malaria-helminth co-infection is the most appropriate term that can be used in the title to capture the mixed infections involving malaria, soil transmitted helminth and schistosomiasis. Furthermore, in lines 120-123, we clearly stated the reason for starting with individual infection involving malaria, STH or schistosomiasis before proceeding with the subject of co-infections. We wrote:* *Although, our primary objective was to ascertain what the caregivers perceived about malaria-helminth co-infection, however, to ensure clarity of the primary objective, we needed first to systematically consider what they perceived to be the risks for malaria and helminths before exploring their perceptions on the co-infection involving malaria and helminths.*

*Therefore, leaving out co-infection or trimming down the title as suggested by the reviewer will completely distort and misrepresent the study findings.*

*Abstract:*

1. Avoid using the word "worms" specify whether it is STHs or tissue-dwelling helminths being referred to in the article.

*Response: We have revised the sentence to read as follows: The parasites causing malaria, soil-transmitted helminthiasis and schistosomiasis frequently coexist…….*

2. Rephrase Ln 24 - 26; to bring out the congruency/co-endemicity of malaria parasites and helminths.

*Response: We have revised lines 24-26 as follows: The parasites causing malaria, soil-transmitted helminthiasis and schistosomiasis frequently co-exist in children living in low-and middle-income countries, where existing vertical control programmes for the control of these diseases are not operating at optimal levels*

3. The aim focuses on co-infections, does it mean that mono-infections have been excluded from the study? Since they appear on the title.

*Reason: As explained above, the primary objective of the study focused on co-infection, but introducing the subject of co-infection involving two or more diseases may be confusing to the study participants, hence, we started with gaining insight about the perception of the study participants about individual infection before introducing the co-infection to the study participants. These were all laid out systematically in the interview guide that we used to implement this study. A copy of the interview guide was also provided to the reviewer for their perusal.*

4. What is the experimental design used? The inclusion criteria used in the study to recruit participants?

*Response: This study used a qualitative design and NOT an experimental design, in whatever forms. The qualitative methods that we used to implement this study including the sampling strategy and how the study participants were recruited in line with acceptable concepts of conducting qualitative research, were adequately described in lines 150-203*

5. In LN 36 "a" before majority.

*Response: We already included the article ‘a’ before ‘majority’ in line 36 of the manuscript that we submitted for this review.*

6. The study findings are ambiguous, How did the author measure perception? Refer to Ln 36 and 37 where the author indicates that the major study finding was that there was a high level of perception. How did they quantify/qualify this?

*Response: We strongly disagree with the reviewer’s comment about the ambiguity of this study. It may interest the reviewer to know that ‘perception’ is an abstract construct that can only be appropriately assessed through qualitative methods which we have used in this study and explained in the methods section of this manuscript. ‘Perception’ is NOT a quantitative variable that can be measured by percentage, proportion or other quantitative methods.*

7. Is awareness different from knowledge? How was awareness determined in the study?

*The concept of ‘awareness’ in social sciences or qualitative research refers to an individual's ability to consider the perspectives of other individuals or on a subject, and apply that understanding to interactions with other individuals or subject. Awareness transcends beyond knowledge. Again, awareness is an abstract construct that can be only be appropriately assessed through qualitative methods such as the interviews that we carefully conducted with a purpose-designed interview guide which exhaustively explored the awareness of the study participants about the study objectives using various qualitative themes. The qualitative interviews were further complemented with participant observations where first-hand assessment of ‘actual’ awareness (not participant’s self-report) about the study objectives were performed.*

8. Ln 39 to 41 is vague.

*Response: Again, we disagree with the reviewer’s comment as this sentence accurately captured the responses obtained from the study participants.*

9. The conclusion is not convincing. Let the author focus on sensitization and health promotion as key study recommendation.

*Response: We do not agree with the reviewer’s comment and suggestion here, because focusing on sensitization and health promotion is an over-simplistic approach to addressing the depth of misconceptions that we observed among the study participants on the subject of co-infection of malaria and helminths. In social sciences, effective interventions are co-created by researchers and the community affected by a problem. This approach ensures adequate uptake of the intervention by the end-users (i.e. the community people and stakeholders). The general assumption that sensitization and health promotion are the answer to all problems identified in a rural setting always fail woefully to achieve the desired results because of poor community engagement/involvement in its conceptualization and implementation.*

*Background Information.*

1. The author fails to establish the niche and eventually occupying it in the study.

Overall, The narrative is anchored on the subjectivity of the author, there is no supporting evidence to make solid conclusions*.*

*Response: The background information provided in this manuscript laid a very strong argument on the need for an integrated control approach for neglected tropical diseases, in line with the recommendation of the 2021-2030 WHO roadmap to end NTDs. Given that availability of reliable data on caregivers’ perception of the risk for malaria-helminth co-infection is key to successful implementation and uptake of coordinated control approaches by the caregivers of children who are at risk of malaria-helminth co-infections, we conducted this qualitative study using acceptable qualitative methods to obtain unique insights and perspectives from the caregivers of the children who are most at risk of malaria-helminth co-infection. The findings of our study were comprehensively described in the manuscript which unfortunately the reviewer failed to appreciate. We hereby re-affirm that the background information, methods, results and discussion in our manuscript coherently reported valid findings about the misconceptions that may negatively affect the uptake of the integrated control approach to malaria-helminth co-infections. Our findings are therefore crucial for planning and implementation of the integrated control programmes for malaria-helminth co-infection.*

Reviewer #2: See comment on the manuscript attached for minor corrections.

*Response: We added lines 219-247 following the explicit request from the editorial team to discuss in the manuscript the study trustworthiness based on credibility, dependability, confirmability, transferability and reflexivity themes. It therefore sounds a bit strange that the paragraphs addressing the editorial queries are being requested to be removed from the manuscript. Also, adding the initials of authors who carried specific tasks in these sentences is an acceptable style of scientific writing, as it reinforces the study credibility that the specific study procedures were performed by the identified member(s) of the study team, hence, we do not agree with the suggestion of the reviewer to remove the initials of the authors from the sentences.*

*Line 254: We have added ‘no’ before ‘risk’ in the sentence*

*Line 256: We have moved the sentence ‘we obtained verbal consent from all potential caregivers before the interviews were conducted’ to line 256 to reads as follows: The Ethics Committees approved the verbal consent procedure for this study; and we obtained verbal consent from all potential caregivers before the interviews were conducted*
